# The EPOS multi-disciplinary Data Portal for integrated access to solid Earth science datasets

**DOI:** 10.1038/s41597-023-02697-9

**Published:** 2023-11-08

**Authors:** Daniele Bailo, Rossana Paciello, Jan Michalek, Massimo Cocco, Carmela Freda, Keith Jeffery, Kuvvet Atakan

**Affiliations:** 1 Istituto Nazionale di Geofisica e Vulcanologia, Rome, Italy; 2 Universitetet i Bergen (UiB), 5020 Bergen, Norway; 3 European Plate Observing System, EPOS ERIC Rome, Italy; 4 Keith G Jeffery Consultants, Faringdon, UK

**Keywords:** Solid Earth sciences, Databases, Environmental sciences, Research management

## Abstract

The European Plate Observing System (EPOS) is a long-term initiative aimed at integrating research infrastructures for solid Earth science in Europe. EPOS provides a sustainable, multidisciplinary user-oriented platform - the EPOS Data Portal - that facilitates data integration, access, use, and re-use, while adhering to the FAIR principles. The paper describes the key governance, community building, and technical aspects for achieving multidisciplinary data integration through the portal. It also outlines the key portal features for aggregating approximately 250 data sources from more than ten different scientific communities. The main architectural concepts underpinning the portal, namely the rich-metadata, the service-driven data provision, and the usage of semantics, are outlined. The paper discusses the challenges encountered during the creation of the portal, describes the community engagement process, and highlights the benefits to the scientific community and society. Future work includes expanding portal functionalities to include data analysis, processing, and visualization and releasing the portal as an open-source software package.

## Introduction

Solid Earth science refers to the study of the planet’s solid surface and its interior, being a component of Earth sciences including geophysics, geology, geodesy and geochemistry. The relevance of solid Earth science extends beyond academic research, influencing human aspirations in areas such as energy, minerals, water, hazards, engineering, and more. Progress in the understanding of the physical processes controlling earthquakes, volcanic eruptions and tsunamis as well as those driving tectonics and Earth surface dynamics requires a long-term plan to facilitate integrated use of data, models and facilities from distributed research infrastructures for solid Earth science. The European Plate Observing System (EPOS, www.epos-eu.org) represents such a long-term plan for the integration of research infrastructures for solid Earth science in Europe. EPOS brings together European research infrastructures and their associated data and services together with the scientific expertise into one integrated delivery system. By improving and facilitating the integration, access, use, and re-use of data, data products, services and facilities EPOS developed a holistic, sustainable, multidisciplinary research platform to provide coordinated access to harmonized and quality-controlled data from diverse Earth science disciplines, together with tools for their use in analysis and modelling. EPOS, relying on new e-science solutions, gives open access to solid Earth data enabling a step change in multidisciplinary scientific research in many, diverse solid Earth disciplines.

Earth scientists have a long-lasting tradition in data acquisition, collection, quality-control and standardization of data and metadata. They are also the key actors for feeding and implementing metadata and services for qualification, storage and accessibility. Research infrastructures, in turn, provide facilities and resources to ensure the data management and interoperability through e-science innovation. Integrating research infrastructures is strategic to tackle the challenge of long-term sustainability from a technical, legal, governance and financial point of view.

The Open Science agenda contains the ambition to make FAIR principles (Findable, Accessible, Interoperable and Re-usable) the basic standard for scientific research^1^. In this framework, data FAIRness is considered a necessary target for research infrastructures in different scientific domains and at global level. The FAIR principles create the conditions to foster data sharing and improve data stewardship, provided that several normative, organizational, and ethical issues are addressed. Research infrastructures have the responsibility to respond to these expectations and fill the current existing gap between FAIR principles and viable practices to FAIRness.

In this paper, we present and discuss the EPOS Data Portal, a user-oriented platform to access, use, reuse solid Earth science data, by describing the key governance, community building and technical elements to achieve the integration of multidisciplinary data in the Solid Earth Science domain. The development of the portal has been achieved through a co-design approach joining skills and experiences of Earth scientists and e-scientists working in the same research environment represented by the EPOS research infrastructure. A pan-European infrastructure such EPOS represents indeed the collaborative framework where overcoming difficulties and sharing solutions to make solid Earth science data accessible and interoperable with the goal of fostering open science.

## Results

### FAIR Data portal for integration of multidisciplinary resources

The EPOS Delivery Framework can be defined as an international, federated framework encompassing the data and services provision integrated from the Thematic Communities (TCS – thematic Core Services) and made interoperable with the central hub of the Integrated Core Services (ICS-C), the novel e-infrastructure for enabling FAIR data management and access^2,3^.

All assets provided by the TCS and integrated within the ICS-C are accessible and available through the EPOS Data Portal (https://www.ics-c.epos-eu.org/).

Such a portal has been designed following a hybrid top-down bottom-up approach: on one hand, the IT architects worked in the different phases of EPOS projects (EPOS Preparatory Phase 2010–2014 and EPOS Implementation Phase 2014–2019) to provide a flexible software architecture for data integration (top-down); on the other hand, the scientific users of the platform were involved through a) Requirement and Use case collection process in EPOS-IP^4^, and b) User Feedback Groups (UFGs) sessions that provided constant and structured feedback during the implementation process of the TCS-ICS integration and the development of the ICS^4^ (bottom-up).

The above led to the definition of the main functionalities of EPOS Data Portal. In order to ensure that the access to community specific assets (data, data products, software and services - DDSS) was compliant with the FAIR principles, a methodology has been established and followed through the entire Software Development Life Cycle of the EPOS Data Portal^5^.

The portal provides access to a heterogeneity of DDSS from ten different disciplines organized in communities, as shown in Table 1 which also reports the specific DDSS provided by these communities.

**Table 1:** List of all Services integrated into the EPOS platform and accessible from the EPOS Data Portal.

Service Group Name	# Services	Data Processing Level
***TCS Seismology***		
Waveform and peak-motion services	42	L0
Seismological products services	10	L1-L2
Earthquake hazard and risk services	11	L2-L3
***TCS Near Fault Observations***		
Seismological data	27	L0-L1
Geochemical data	9	L0
Geophysical data	4	L0
***TCS GNSS Data and Products***		
Data	3	L0
Products	10	L1-L2
***TCS Volcano Observations***		
Seismic data	6	L0
Geodetic data	7	L0
Geochemical data	1	L2
Satellite data	1	L1-L2
Ground-based remote sensing	3	L0-L1
Volcanological/petrological data	9	L1-L2
Geohazards	9	L1-L3
***TCS Satellite Data***		
InSAR	8	L1-L2
***TCS Geomagnetic Observations***		
Geomagnetic data	7	L0-L1
Geomagnetic models	2	L2
Geomagnetic indices and events	4	L1
Magnetotelluric models and data	4	L1-L2
***TCS Anthropogenic Hazards***		
Services	2	L1
Episodes	39	L0-L2
***TCS Geological Information and Modelling***		
Boreholes	2	L2
Geological maps	2	L3
3D/4D models	2	L2
Mineral resources	2	L2
***TCS Multi-scale Laboratories***		
Analogue modelling of geologic processes	1	L2
Rock and melt physics	1	L2
Paleomagnetism	1	L2
Geochemistry	1	L2
Microscopy and tomography	1	L2
Cross-discipline laboratory data	1	L2
***TCS Tsunami***		
Tsunami data	1	L1
Hazard and risk products	8	L2-L3
Tsunami catalogue	10	L2
Submarine landslide catalogue	6	L2

For each service category, the table reports the domain, the services name and the number of Services of that type integrated, together with the DPL.

Services give access to data from various data providers geographically distributed over the entire European Lithospheric Plate.

The more than 250 services, deliver more than 30 different types of data – spanning for instance from miniSeed waveforms for Seismology to tiff images for Satellite Data and pdf reports for Volcanology - described by more than 20 different types of metadata. This represented a real challenge in terms of integration and interoperability^3^.

### Data provision

In order to understand how to interoperate the data provided by the TCS, these have been categorized into four data processing levels (DPL), with respect to their scientific complexity and amount of post-processing (either automated or human driven):

- *Level 0*: raw data, or basic data (e.g., seismograms, accelerograms, time series). These data represent the output of the sensors or actuators used to measure the physical properties or phenomena.
- *Level 1*: data products coming from nearly automated procedures applied to raw data at level 0 (e.g., earthquake locations, magnitudes, focal mechanism, shakemaps).
- *Level 2*: data products resulting from scientists’ investigations (e.g., crustal models, strain maps, earthquake source models). This data requires the intervention of scientists that interpret and add values to Level 0 or Level 1 data. Typically, Level 2 data are scientific results in the form of images, histograms, and graphs included in scientific papers.
- *Level 3*: integrated data products coming from complex analyses or community shared products, which require collaborative processes over a considerable time span, as in the case of hazards maps^6^ or catalogues of seismogenic faults^7^.

Table 1 presents an overview of the Services providing access to community-specific datasets together with the data processing level.

Services from the Tsunami community^8^ are currently in the status of “candidate TCS”. It means that they expressed their interest in being part of the EPOS pool of services, and are working for full integration, also considering governance and sustainability dimensions, beyond technical one.

While the EPOS portal currently offers a broad spectrum of services, the importance of continually expanding and refining the portal offerings was recognized and addressed: in response to the evolving needs of the geoscience community, EPOS is actively working to integrate more diverse datasets, and incorporate additional thematic communities, such as the “Built Environment Data Thematic Community,” exemplify EPOS commitment to this expansion.

### The EPOS Data Portal

**Fig. 1:**
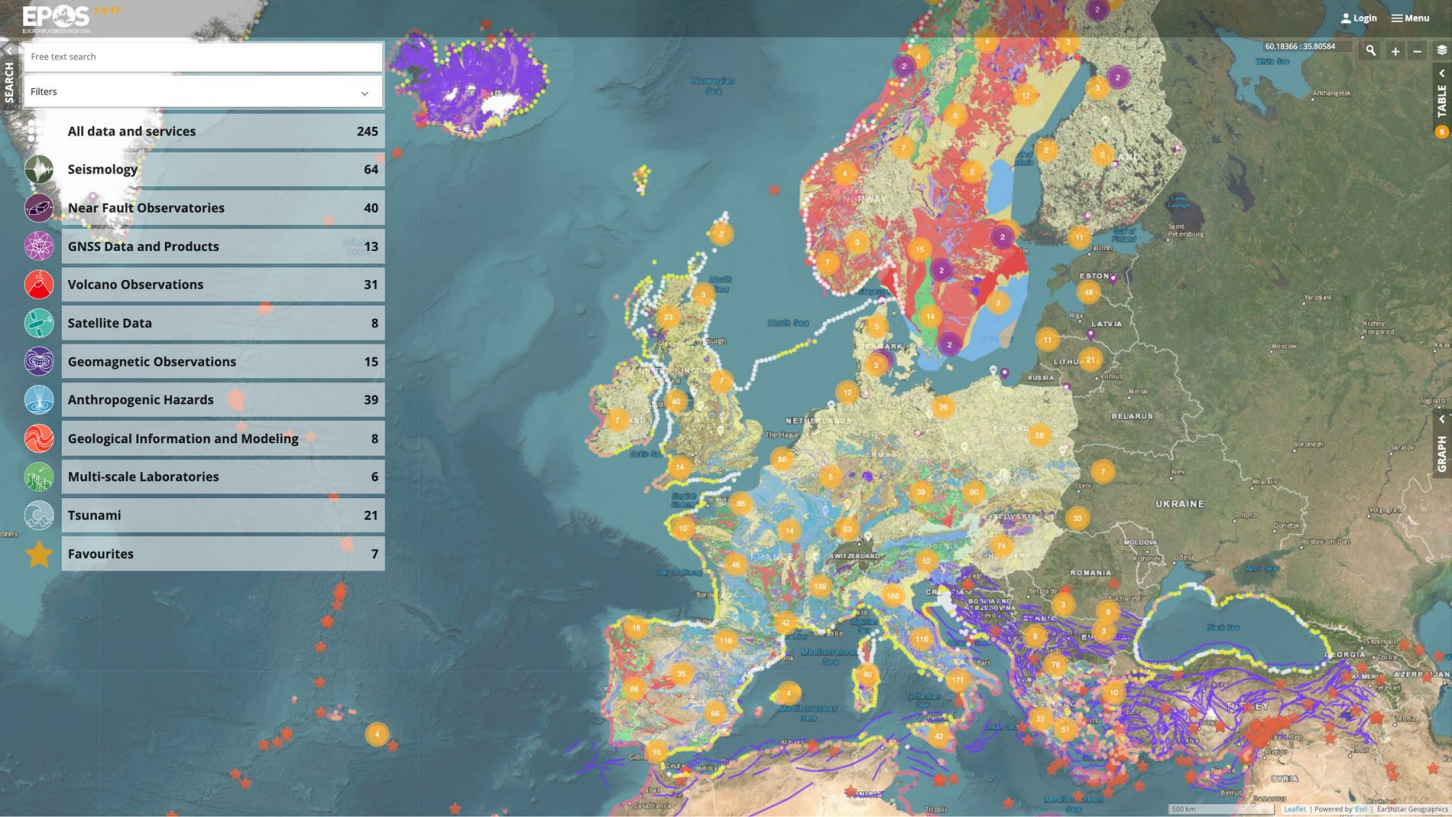
The EPOS Data Portal User interface showing a combined view of different datasets: European Database of Seismogenic Faults - Crustal Faults (blue lines) – offering insights into potential seismic activity zones; GNSS Stations (orange circles) – providing geospatial accuracy and tectonic movement data; Geological Map – revealing the underlying geological structures and formations; magnetometer stations (purple circles)– indicating geomagnetic variations and their potential implications; List of Anthropogenic episodes (white balloons) – cataloguing significant events; Tsunami Hazard Map – highlighting regions at risk from tsunami events based on historical and modeled data; Modern earthquakes (orange stars) – presenting recent seismic activities and their locations.

The EPOS Data Portal is based on a user-friendly user interface (Fig. 1) which provides intuitive visualization methods and interaction modes - compliant with common data portals development guidelines^9^ - that significantly facilitate the discovery and the access to the geoscientific community assets. In addition, recognizing the need for cross-border collaboration, the EPOS Data Portal is engineered to enable users to seamlessly assemble data across different jurisdictions. From end-users’ perspective, the discovery action follows four stages which also emerged in other works^10^: (1) *Search*, by specifying a set of search criteria. (2) *Results exploration*, browsing the retrieved results and pre-visualize them in different ways. (3) *Results refinement*, where results can be further refined. (4) *Final selection*, i.e., downloading the selected results or putting them into a list of favourites.

At the *search* stage, the available assets can be filtered by combining free-text search, spatio-temporal range, data providers and by type of visualization.

At the *results exploration* stage, the aim is to contextualise results, i.e., to validate and evaluate how much each individual result satisfies the requirements. The Data Portal offers three different types of visualization of the results to enhance the decision-making:

1. *Map view*, showing results having associated location information in an interactive map. The map provides basic GIS functionalities (e.g., move, zoom, get information about features by selecting them, customize the markers) and datasets can be visualised individually or overlaid to obtain an integrated view. This is useful, for instance, for geo-referenced data like, e.g., geological maps, maps of seismogenic faults^7^, satellite images (e.g., SAR images), as well as point information, e.g., from seismic events, GNSS stations, etc.
2. *Graph view*, where users can plot results on a 2D graph. It applies to time series which are typical results of continuous measurements done by sensors in various disciplines, such as GNSS Position Time Series^11^.
3. *Table view*, which presents service content in a table where each row represents a result, and columns show the various quantity values. This applies to geolocated data as well as to non-geolocated data, such as PDF files or software.

The map view and table view are linked together for geo-located data allowing users to easily switch between the views.

Supplementary information on each result, namely DDSS, is provided by the Detail view, which includes name, description, license, Persistent Identifiers (e.g., Datacite DOI^12^) and other relevant information.

At the *results refinement* stage, the individual results can be further adjusted through service-specific options that allow users to define higher levels of granularity and find the desired data.

At the *final selection* stage, users can download the results in several formats to their own personal computer or add the results into a favourite area.

An example use case demonstrating the added value of the EPOS Data Portal for searching and aggregating multi-disciplinary data is presented in the Supplementary information.

## Discussion

### Challenges

The creation of the EPOS Data Portal required to address challenges along different dimensions.

Firstly, the community building dimension: one of the lessons learnt in EPOS in the last decade is that addressing the technical aspects alone is not sufficient. To achieve integration, considerable work for building, organizing, and keeping the engagement of the international community of earth scientists, e-scientist, data practitioners and managers is required.

The EPOS community is primarily divided into two main categories: the first one involves the “internal communities” that constitute the entire EPOS delivery framework, which include the data providers at the national, regional, or international level, as well as the 10 thematic communities at a European level. The total number of research organizations involved within the “internal communities” is more than 140, with several hundreds of individuals actively participating.

The second category involves the “external communities”, that are not part of the EPOS delivery framework. These can be grouped into different stakeholder categories, including scientists (main users of the portal), government organizations, private sector and industry, and society at large.

In addition, the development of the Portal is carried out by a developers’ community (mainly IT-specialists but also including domain scientists from the solid Earth Science community). The total number of skilled professionals involved in developments is around 50 individuals.

Managing such heterogeneous communities – internal, external, developers - including engineers, IT Developers, and Scientists, with the common goal of building a data integration portal, is undoubtably a huge challenge that in EPOS is tackled by implementing periodic interaction workshops (the so called “ICS-TCS interactions”). These are coordinated by an interaction team and are organized with a systematic approach, inspired by the “shape-up” method^13^ reviewed for the research context. Each year, four one-week workshops are organized: the developers’ community, the representatives of the community scientists, come together to jointly discuss and agree the key developments to be done in the following development cycle of 3-months duration. As such, there are 4 ICS-TCS workshops followed by 4 development cycles of 3-months duration.

The entire work is coordinated by the ICS-TCS Interactions Coordinator and is governed by an IT-Board which includes the key members of the EPOS governance such as the IT Officer, Developers, Operators, Data providers and others. The IT-Board prepares annual plans based on the community requirements and on the strategic considerations as expressed in the EPOS strategic documents. These plans, after being reviewed and endorsed by the EPOS Coordination Office and the Communities, represent the backbone of the smaller granularity activities managed through the interaction workshops.

The second key dimension in which considerable challenges had to be tackled is the technical dimension. Five key challenges were addressed here: (1) The creation of a Data Portal user interface for multidisciplinary data access, (2) the design of a scalable and sustainable architecture for the data integration system, (3) the management and integration heterogeneous, rich, community specific metadata standards, (4) the harmonization of different data, metadata formats and protocols used for stewarding data and (5) the compliancy to FAIR principles.

At the Data Portal level, the technical challenges were addressed with a threefold approach: on one hand, the integration system (Integrated Core Services Central Hub - ICS-C) adopted a *service-based architectural approach*, implementing modularity, enabling scalability and allowing interoperability with underlying web services (APIs) provided by the Thematic Communities of data providers; on the other hand, such system adopted a *metadata model* (and associated catalogue) allowing the description of concepts of interest by the communities in a formal and canonical language; finally, the usage of *semantics attached to metadata*, described by the communities with a common vocabulary and serialized in a machine-readable format, enabled to harmonize both the queries and the responses to/from the data providers nodes^14^. These approaches are further described in detail in the Methods section.

At the architectural level, the challenge was designing a scalable and sustainable integration system with the capability of evolving over time as new requirements are elicited and as more DDSS are integrated. The adoption of a design inspired to the Microservice approach^15^ and based on a rich metadata and services driven paradigm^3,14^, enabled the provision of a flexible, scalable and FAIR compliant architecture. Noteworthy, the combined usage of these three elements allowed EPOS to adopt a paradigm which, differently from the warehouse model, provides access to the digital assets without the need of storing them into the Integrated Core Services Hub, as all conversions are done run-time.

At metadata level, the challenge was represented by richness of the metadata, one of the key FAIR subprinciples, required for implementing Findability, Reusability, and Interoperability. Metadata, indeed, are used to describe the various DDSS assets in a rich and machine-readable way, including details enabling to access and query the data providers web-services nodes. This is technically done by using: a) EPOS-DCAT-AP^16,17^, an extension of DCAT-AP^18^, which allows communities to describe in a relatively easy, standard, and rich way their services; b) EPOS metadata catalogue^19^, which is used to store in a rich way all information about the various DDSS. The metadata catalogue is based on a refactored version of the CERIF model^20^, a rich metadata superset with a formal syntax and declared semantics, from which it is possible to extract metadata in different formats (e.g., Dublin Core^21^). A procedure for mapping from EPOS-DCAT-AP to the metadata catalogue was established to preserve the metadata richness, as better described in the Method section.

At the level of data provision, a technically relevant challenge addressed is also the harmonization of different data, metadata formats and protocols used for stewarding data. This process, undertaken also in other initiatives like ESS-DIVE^22,23^, implied a commitment by all communities for adopting common standard-based (meta)data structures, formats, and communication protocols. It included a semantic harmonization - an ongoing work for terms definition, harmonization, and cross-linking within and across multi-disciplinary communities.

To support and sustain the EPOS communities in the metadata harmonization and population processes, collaborative tools have been especially helpful. In addition to common tools like GitLab, GitHub, an ad hoc Metadata Editor named SHAPEness^24^, was developed and adopted. Such activities guarantee a harmonized data provision through the EPOS Data Portal, and also represent an added value for the communities adopting standards, thus increasing their FAIR compliancy.

Finally, for tackling the FAIR compliancy challenges, specific task forces were set up for tackling key topics like Authentication, Authorization and Accounting Infrastructure (AAAI), Persistent Identifier (PID) adoption, Vocabulary and Semantics. This guarantees that the FAIR principles are considered in the overall development of the integration system underpinning the EPOS Data Portal.

### Added value

Geoscientists play a key role in society, offering knowledge that spans from conceptual research to informed management. The EPOS Data Portal is designed to support these multifaceted contributions, particularly in the realms of mapping (spatal dimension) and monitoring (temporal dimension). As a consequence, the endeavours for building the EPOS multidisciplinary Data Portal have brought significant advantages to the Solid Earth community, to the environmental cluster and, in a longer term perspective, to the entire society by facilitating a multi-faceted and multi disciplinary approach to complex scientific, societal and environmental problems.

The first and most apparent added value is the availability of a one-stop-shop portal that enables any user to access Solid Earth data in an integrated way. Notably, all data is available from the portal itself, and redirection to the discipline specific services, websites or portals was not encouraged, although necessary for some very specific datasets. In addition, dealing with one single portal requires learning the usage of one interface only instead of many (one for each data provider’s data), thus smoothing the usually steep learning curve for such features-rich portals. It is obvious that the Data Portal cannot satisfy requirements on functionality from all communities. The current features were designed to allow data discoverability and selected visualizations. However, the development is not over, and more is described in section Future work.

Another added value concerns the interaction among the different dimensions: for ensuring a robust, continuous, and sustainable provision of the data, an entire supporting governance structure had to be designed and implemented on top of the technical infrastructure. A brief description has been provided above, more details are reported in other works^25,26^ where the EPOS governance framework is described in detail.

Consequently, EPOS represented an added value not only for the scientific community in general, but also, and perhaps primarily, for the domain specific communities committed to data provision. These had indeed to secure or create an entire governance structure which contributed to the community building and to the internal harmonization in terms of technical, financial, and governance practices. A virtuous example is the GNSS community, that built a new European data access hub (GLASS portal^27^) capitalizing previous efforts in EUREF, the, Permanent GNSS Network that consists of a network of continuously operating GNSS (Global Navigation Satellite Systems, such as GPS, GLONASS, Galileo, Beidou, and others) reference stations, and other assets (http://www.euref.eu/). This required all National networks and data centres to converge towards common technologies, protocols, and governance practices.

Beyond the Solid Earth science domain, EPOS represented an added value as reference and technology provider for other contexts. This is the case for instance of the ENVRI-FAIR initiative^28^, where the EPOS metadata catalogue software was adopted for providing a machine-readable interface towards the European Open Science Cloud portal (https://eosc-portal.eu/). Similarly, the JERICO initiative^29^ (represented by the current JERICO-S3 project, available at https://www.jerico-ri.eu/projects/jerico-s3/) considered the adoption of the EPOS architecture for providing software services to their users.

Finally, the usage of solid Earth science data for geo-hazards assessment and risk mitigation and the engagement of the data and service providers formally committed to carry out the surveillance of the national territory from natural and anthropogenic events, are clear added value for the entire society. This raises the need to consider also the ethical dimension associated with the Solid Earth data provision, as discussed elsewhere^30^.

The above-mentioned challenges and added values of EPOS also emphasize the key role of Research Infrastructures, which are indeed the place where, working across different dimensions (technical, scientific, legal and governance), FAIR principles and practices are implemented in reality^30^ thanks to the adoption of a co-development approach oriented to Open Data, and a strong technical harmonization action across communities of developers and data providers.

### Future work

The EPOS Data Portal shows the features of a system designed to provide integrated access to multidisciplinary data, guaranteeing both a robust technical, FAIR compliant data provision and the governance and financial sustainability in the long term.

However, the EPOS long-term perspective accounts also for evolution of the research methods and practices which require to go beyond the integrated data access: requirements converge towards the need for environments where also data analysis, processing and visualization can be carried out, and ideally in a reproducible way. This is indeed already a practice in many communities that make use of interactive cloud environments^31^ like Jupyter Notebooks^32^, with complementary data-management and data-preparation services.

The future work will therefore focus on the integration of Distributed Integrated Core Services (ICS-D), a concept already discussed in EPOS, i.e., virtualized computing facilities to support research data analysis, workflow management and notebook management. This will enable the EPOS Data Portal to become a fully effective Virtual Research Environments (VREs)^33^ covering the data Life Cycle almost entirely. The option of sharing the workflow either with other researchers in a research team or even publicly can speed up finding solutions to the problem and also give transparent insights to the used method and allow reproducibility.

This poses interesting challenges in several dimension: firstly, all resources need to be described properly by means of metadata, thus potentially requiring a further evolution of the EPOS-DCAT-AP model for including additional metadata elements; then, to follow the standard-based approach, common interfaces for communicating with cloud resources should be selected and developed. Preliminary work on this matter has been done, showing that APIs based approach using SWIRRL^31^ allows VREs like the ICS-C Data Portal to easily integrate external cloud resources or tools like Jupiter notebooks, or, in the case of EPOS, the Enlighten-web^34^ visualization tool.

Future work includes improvement of architectural modules like the convertors, to widen the integration to new types of data formats.

A key future activity is also the provision of the EPOS system as an open-source package, so to enable other communities to capitalize the work done within EPOS under the umbrella of the European Commission. A preliminary release is available at https://epos-eu.github.io/epos-open-source/.

Finally, EPOS will continue collaborating with a cluster of research infrastructures concerned with the environment (ENVRI)^28^ and with the European Open Science Cloud (EOSC)^35^, which will offer opportunities for increased computing power, large-scale storage management and interoperability across clusters. In addition, EPOS staff will continue contributing actively to discussions at RDA (Research Data Alliance, https://rd-alliance.org/), IEEE Big Data management group (https://bigdata.ieee.org/) and other fora.

## Methods

### EPOS delivery framework

**Fig. 2:**
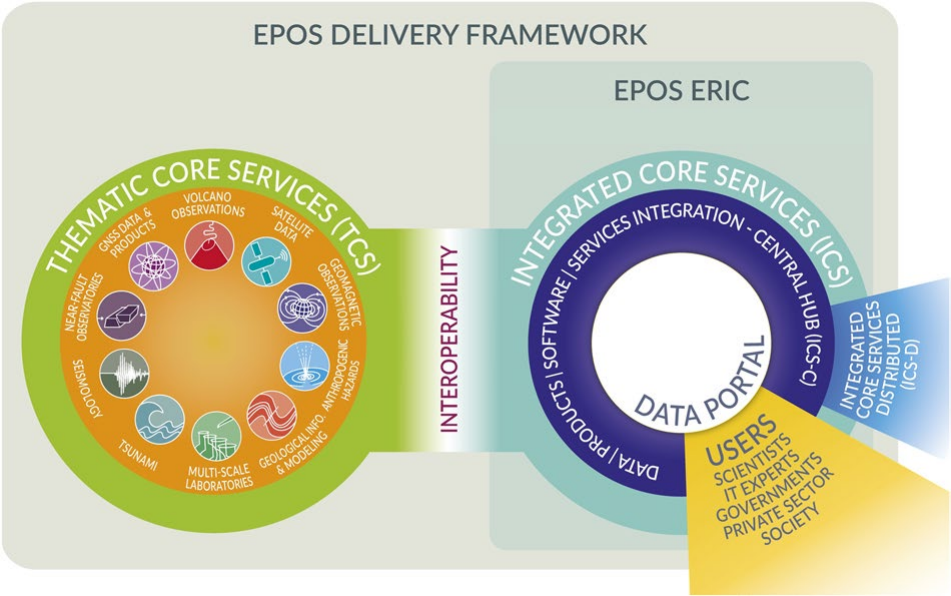
Main elements of the EPOS functional Architecture: Thematic Core Services, Integrated Core Services Central Hub (ICS-C) and Integrated Cores Services Distributed (ICS-D). These are coordinated by EPOS ERIC and designed to ensure the EPOS Data and service provision.

The functional architecture underlying the EPOS Data Portal includes three main high-level components (Fig. 2) that compose the so-called EPOS delivery framework.

The first one is represented by the Thematic Core Services (TCS), discipline specific nodes that enable the integration of data and services from domain communities. The technical architecture of the services provided by the individual communities is not prescribed, but metadata describing the interface (APIs or web services) for accessing the assets need to be provided in a common knowledge language which enables the interoperability with the Integrated Core Services Central Hub (ICS-C). The ICS-C, the second component of the framework, is the technical node where integration of data, metadata, services, and distributed computing occurs. The Data Portal described in the current work is the entry point to access resources integrated by the ICS-C. The third component is represented by the Integrated Core Services Distributed (ICS-D). The ICS-D concept is to extend the ICS-C in a virtualized way with additional computing facilities to support research data analysis, workflow management and notebook management. This component is currently under development, but prototypes connecting ICS-C integrated resources with Jupyter notebooks were released and look promising^31^.

Users accessing the data from the ICS-C were categorized into different user categories (e.g., Scientists, Governments, private sector, and others). The requirements and expectations from different user groups indeed influence the system developments and functionalities and are strictly connected with the way the data is provided and shown on the Data Portal. Currently the Portal addresses mostly the Scientific users target group.

**Fig. 3:**
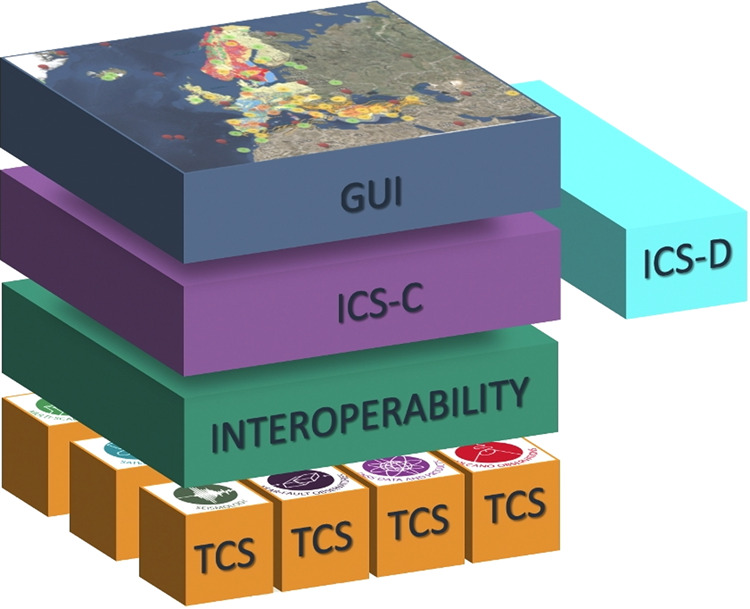
The EPOS technical architecture consists of four tiers, Thematic Core Services (TCS), Interoperability, Integrated Core Services (Central Hub and Distributed), Graphic User Interface (GUI).

The generic architecture just described in Fig. 2 shows the different high-level components and their mutual interaction. The technical architecture in Fig. 3 is complementary and explains more in detail the software e-Infrastructure supporting the EPOS Data Portal.

### EPOS Data portal technical architecture

The EPOS technical architecture (Fig. 3) is represented as a cube that implements a four-tier architecture. A detailed description of each of the components follows in a bottom-up manner.

#### Thematic Core Services (TCS)

Each community provides access to its resources through European-wide services called Thematic Core Services (TCS). TCS are characterized by enormous heterogeneity of data types and formats, protocols for accessing data and metadata, and scientific methods. Therefore, a huge amount of work for harmonizing data and metadata has been done both at TCS level and across TCS. TCS provide access to (meta) data and data products by means of machine actionable and machine-readable Web Services APIs.

TCS are designed to be FAIR compliant, and the “FAIRification” process is part of the governance and data provision agreement that each community has committed to.

#### Interoperability Layer

The interoperability layer was created to exchange metadata between TCS and ICS. The design of such a layer was done by considering communities requirements, without imposing a unique metadata schema, vocabulary, or technical approach, but, at the same time, adopting a strong and robust procedure to achieve data integration. The common knowledge representation underpinning the layer was defined in the form of a metadata application profile and called EPOS-DCAT-AP^16^, as it is an extension of the Data Catalog Vocabulary Application Profile (DCAT-AP). It is used to capture, organise, and harmonise information from different sources and to enable semantic interoperability between the two layers (TCS and ICS). Information about communities’ assets and the way how they are accessible are described through metadata descriptions that are then stored into the central catalogue within the Integrated Core Services.

#### Integrated core services – central hub system (ICS-C)

The ICS-C is the place where the integration of data, data products and services occurs. It is based on a microservice oriented architecture and designed to maximize the portability, scalability, and security of the system. The microservices paradigm, indeed, enables the system to a) run in a distributed environment based on Kubernetes; b) adopt a software-independent approach ensuring up-to-date technological upgrades; c) properly scale specific system functionalities so to ensure maximum availability, d) enhance reliability; e) isolate independent software applications running in a shared environment, e.g., by using Docker.

A set of *WEB APIs* allows to consume and manipulate metadata information stored into the metadata catalogue based on a refactored version of CERIF (Common European Research Information Format)^20,36^. The Graphic User Interface (GUI) interacts with WEB APIs in order to provide users several features like discovery, contextualising, downloading.

#### Integrated core services – distributed

The implementation of the ICS-D makes EPOS a true multi-purpose Virtual Research Environment covering the Data Lifecycle from data production to data reuse and further data analysis for the creation of new data products. Currently, two prototypes have been developed: Enlighten Web and Jupyter through the usage of the SWIRRL APIs^31^. The former is an advanced online tool for visual analytics of geospatial datasets, while the latter offers the well-known functionalities of a literate programming environment, with additional reproducibility controls, and integrated data & software management capabilities. The two tools can be used in combination, sharing data and workflows in a fully integrated dataspace, in the cloud. Currently, the level of integration with the ICS-C is achieved at the prototype level only and hence not yet accessible from the operational instance of the EPOS Data Portal.

#### Graphic user interface (GUI)

Data, data products, software and services integrated within the ICS-C can be accessed by means of the Graphic User Interface (http://ics-c.epos-eu.org/). It represents the front-end of the whole system which provides a uniform access to a plethora of data available in a very heterogeneous way. It is a map-based interface which combines an intuitive, user-friendly interface with multiple features including browsing, visualizing, and contextualizing a diverse geoscience dataset, as well as a guided tour of the key features. The interface was implemented in an iterative and user-oriented design process. Various research methods were used in this process, such as sketches, mock-ups, personas and above all user tests to assess the user experience and usability of the portal as described in the following section. In addition, a feedback web form is available for everyone within the portal GUI to report bugs or comments at any time.

### Data portal system development

The Data Portal System follows a FAIR Data management approach^3^ and has been developed using a combination of open-source technologies for each of the architecture layers. These include Java, RabbitMQ, python and others. We have implemented a modular architecture based on the microservices paradigm^15^, that allows for easy integration of new data and services through dedicated software interfaces. A detailed technical documentation of the system is available in the online developer’s guide attached to the open source code preliminary release (https://epos-eu.github.io/epos-open-source/).

Datasets from the thematic providers are made available and accessible through standardized web services. Such services are described by means of a common representation language – namely EPOS-DCAT-AP^16^ – which represent the common metadata standard for providing information to the central integration system underpinning the Data Portal. The addition of new datasets or services is managed dynamically with the metadata description in EPOS-DCAT-AP.

The metadata are stored centrally in a catalogue^20^, which enables the system to manage the wealth of different resources through an approach that uses in a combined way metadata, semantics, and web services to implement the interoperability^14^.

Following such an approach the heterogenous thematic data are made available in a harmonised way and presented via RESTful APIs which are in turn consumed by the user interface of the Data Portal.

## User feedback

The EPOS Data Portal has been tested and evaluated by multiple groups of users. User Feedback Groups (UFG) had been established in each thematic community during 2016–2019 and the members have participated in testing of the portal several times (two workshops in 2019). The Pilot Operational Testing phase (2020–2022) consisted of testing of the whole EPOS system and user testing of the portal was arranged in June 2021 for two groups of users: general users and scientific users. This user testing was repeated in June 2022. All the groups of users were providing valuable feedback during testing and not only reporting issues but also suggesting new features. There was an interesting observation coming out from the scientific user testing when the participants were asked to visit dedicated thematic community portals and perform similar tasks there. These portals are focused on their own community needs having more advanced features allowing deeper analysis of the datasets. It is significant that most user testing participants preferred using the EPOS Data Portal.

The feedback can be provided by any user directly through the Feedback link on the portal. The feedback goes to EPOS GitLab repository where the issues are evaluated and managed.

### Supplementary information

Supplementary Information

## Data Availability

The current work did not produce any new dataset. All data available from the EPOS Data Portal (https://www.ics-c.epos-eu.org/) is provided by the data providers who distribute their data at their premises with their own services, technologies and agreements. Availability of data through the portal is ruled by dedicated Data Provision agreements between EPOS ERIC and the data providers where data providers agree that to facilitate effective rights and ownership management, the DDSS Licence shall be compliant with the EPOS Data Policy and thus aim to adopt a Creative Commons 4.0. licence (CC:BY and CC:BY:NC). Where for relevant reasons a licence cannot be applied, the Coordinator and their Participants shall inform EPOS ERIC. All references (e.g., URL and DOIs) to data providers portal services and datasets, are available in the data portal “information” area of each Dataset or Service.

## Code availability

The code developed for the EPOS Data Portal system is available on the GitHub repository of EPOS https://epos-eu.github.io/epos-open-source/. Other code that contributed to the development of the system is already available on such repository, as in the case of the SHAPEness metadata Editor (https://github.com/epos-eu/SHAPEness-Metadata-Editor) and the specifications for EPOS-DCAT-AP extension (https://github.com/epos-eu/EPOS-DCAT-AP).
