# Supplementary material for: The EPOS multi-disciplinary Data Portal for integrated access to solid Earth science datasets
Source: Sci Data. 2023 Nov 8;10:784. doi: 10.1038/s41597-023-02697-9 (PMC10632364; doi:10.1038/s41597-023-02697-9)
Supplement: Supplementary file 1 — Supplementary Information [file 41597_2023_2697_MOESM1_ESM.pdf]

Supplementary information

Using EPOS Data Portal

Main audience of the EPOS Data Portal are cross-disciplinary geoscientists reaching out to new scientific areas outside of their own specialization with the aim to find and understand the new possible linkage between datasets. Making this multi-disciplinary approach possible requires integration of datasets with similar levels of details which would allow contextualization of information across domains. The EPOS Data Portal is not currently able to provide advanced data analysis tools but serves for data discovery and contextualization across geoscientific domains. Users can explore the datasets and decide whether there are datasets suitable for their research. The multi-disciplinarity also introduces a new paradigm and expectation of wide and possibly deep research knowledge and might require additional training.

To demonstrate the current capabilities of the EPOS Data Portal we have chosen one specific scientific use case. Detailed steps are described in Table S1. This scientific use case is covering ten different services/datasets from six different thematic communities. Such simultaneous data exploration is not possible in any other system yet.

The datasets in which the user is interested in can be saved as favourites and then can be visualized together on the map. User interface with all datasets used in the scientific use case are (see Table S1) shown in Figure S1. Each dataset in the list of selected items is plotted on the map with markers of different colours and also visible in the table view (Figure S2).

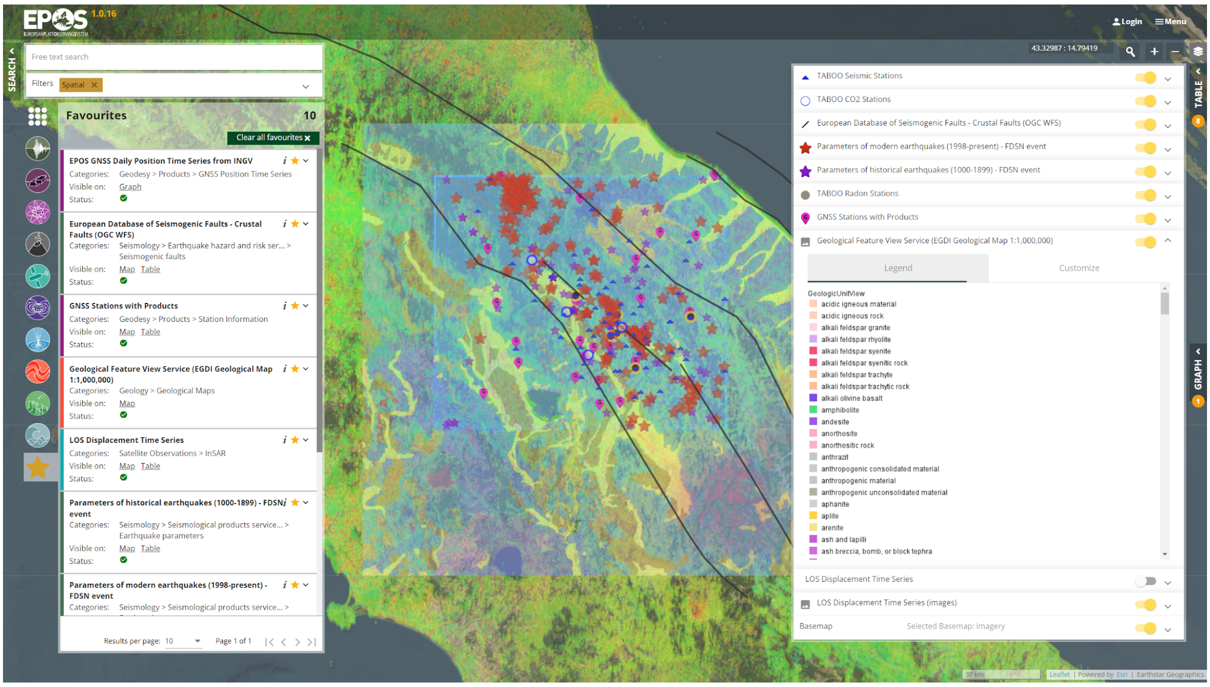

Figure S1: Map view in EPOS Data Portal with selected datasets.

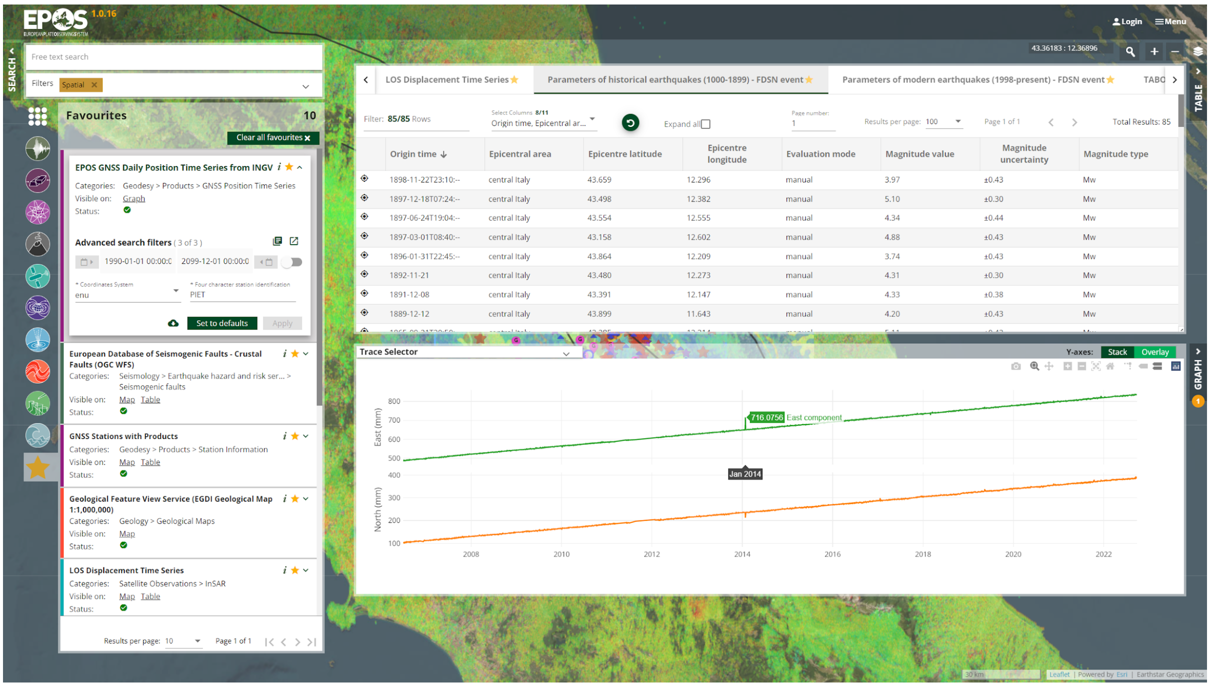

Figure S2: Table view and graph view in EPOS Data Portal. Exploration of individual datasets in table view is possible by clicking the tabs above the table. Graph view is showing horizontal GNSS time series from station PIET.

Graph view is currently supported for time series in covJSON format but there is planning to support more 2D graph data to be visualized here.

Table S1:

**Scientific User Story**	
As a researcher, you are investigating a major fault zone that is monitored by a range of scientific instruments, including seismic, geodetic, geochemical and other stations. Your main goal is to find transient signals that may be related to the activity of the fault. Below, we provide you with some basic tasks that will help you to explore the EPOS Data Portal.<br>We suggest that you focus on the Alto Tiberina near-fault observatory (TABOO - central Italy).	
**Task**	**Action**
1. Find, display and explore seismogenic faults in the TABOO region.	1. To locate TABOO NFO, click the search tool in map view and search for 'Alto Tiberina' in Map view, select the first item in the list and then zoom out until you see W and E coastlines of Italy. Draw a rectangle around this area as a geographical bounding box (tool can be activated in the upper left corner) to limit further results in the search to this area.<br>2. Search for 'faults' in the free text search field in Advanced search. List of services is reduced.<br>3. Navigate to Seismology and select 'European Database of Seismogenic Faults - Crustal Faults (OGC WFS)' service.<br>4. Click the 'pin icon' next to the service to add it to your list of selected items.
2. Find, display and add	1. Search for 'events' in free text search. Filters will limit the

recent earthquakes to your list of selected services. How many earthquakes of M>3 (within depth 40 km) were located around TABOO observatory (43°-44°N, 11.9°-13°E) between 2010 and today?	number of services to ten.<br>2. Navigate to 'NFO -> Seismological Data -> Seismological Products -> Earthquakes data -> TABOO Events'.<br>3. Use the Configuration pane to adjust the date, limit (10 by default) and minimum magnitude parameters.
3. Find and display geological map. Adjust transparency of the map layer so that the seismogenic faults are better visible.	1. Clear advanced search filter.<br>2. Navigate via facets to 'Geology -> Geological maps -> Geological Map 1:1,000,000 (OneGeology-Europe layer)'.<br>3. Open 'Layer Control' in Map view and adjust transparency.
4. Find and display various observing stations in the TABOO region.	1. Search for 'stations' using advanced search filter.<br>2. Navigate via facets in individual thematic facets to find the relevant services for stations.<br>3. Switch to 'Table view' and select the appropriate service to get the number of stations (rows in table).<br>4. Pin all services from questions above (seismic, GNSS, geochemical) into your list.<br>5. Click the download button next to service and choose JSON for downloading the list of stations.
5. Find and display historical earthquakes in the region. What is the largest historical earthquake around the TABOO observatory? What lithology is in the area of that earthquake?	1. Search for 'historical' using advanced search filter.<br>2. Select the 'Historical earthquakes of interest for the TABOO NFO (FDSN-event)' service.<br>3. Switch to 'Table view', select the appropriate service and sort events by magnitude in the table (click the column name, twice).<br>4. Locate earthquake in map by clicking the 'Show on map' cross-hair icon in the table. Alternatively, copy coordinates of the event from the table 'Epicentre (lat, lon)' and paste it into the search tool in 'Map view'. Add 'N' after latitude and 'E' after longitude -> search. Map will zoom to that event.<br>5. Enable/adjust 'Geological map' layer to get the colour (wait for the layer being loaded). You might need to disable other layers.
6. Visualize GNSS stations with products in the TABOO region on a map and explore them. Check general horizontal movement of GNSS stations in the TABOO region.	1. Navigate to 'Geodesy -> Products -> Station information -> GNSS Stations with Products'.<br>2. Zoom to TABOO region. Click on the station marker in the map and open a link to the time series image in the pop-up marker label. Explore multiple stations if needed.<br>3. Switch to table view, select 'GNSS Stations with Products', use the filter above the table and search for station ID. Locate station in map by clicking the 'Show on map' cross-hair icon in the table. Map will zoom to that station.<br>4. Switch to service 'EPOS GNSS Daily Position Time Series from INGV' and update the configuration to station of interest. Open the 'Graph view' and add time horizontal time series to plot by clicking the '+' sign. Analyze the time series.
7. Zoom in to the latest earthquake with M>2 in map	1. Search for 'satellite' in advanced search.<br>2. Navigate to 'Satellite Observations -> InSAR -> LOS

view and explore LOS Displacement Time Series (InSAR data). Do you consider the area near that earthquake as stable based on 'LOS Displacement Time Series'?	Displacement Time Series'.<br>3. Green colour indicates stable areas. Other colours indicate uprise or subsidence.
